# Interpolymer Complexes of Poly(methacryloyloxyethyl phosphorylcholine) and Polyacids

**DOI:** 10.3390/polym14030407

**Published:** 2022-01-20

**Authors:** Tatiana Nekrasova, Olga Nazarova, Elena Vlasova, Andrei Fischer, Yuliya Zolotova, Marina Bezrukova, Evgeniy Panarin

**Affiliations:** Institute of Macromolecular Compounds, Russian Academy of Sciences, Bolshoi Pr. 31, 199004 St Petersburg, Russia; imc@hq.macro.ru (O.N.); spectra@imc.macro.ru (E.V.); andreasfischer@mail.ru (A.F.); bezrukova@imc.macro.ru (M.B.); lab.2@macro.ru (E.P.)

**Keywords:** poly(2-methacryloyloxyethyl phosphorylcholine), poly(carboxylic acids), poly(vinylphosphonic acid), interpolymer complexes, intramolecuar mobility

## Abstract

It has been shown that macromolecules of poly(methacryloyloxyethyl phosphorylcholine) can form hydrogen bonded interpolymer complexes with homo- and copolymers of carboxylic acids and with poly(vinylphosphonic) acid in aqueous solutions. Polarized luminescence and IR spectroscopy were applied in the investigation. Nanosecond relaxation times characterizing the mobility of the chain fragments for the initial luminescent labeled polymers were determined and their changes by a factor of 2–50 were established during the formation of an interpolymer complex. Hydrogen bonds play a dominant role in the formation of these complexes. Hydrophobic interactions serve as an additional stabilizing factor. It is established that poly(methacryloyloxyethyl phosphorylcholine)/poly(vinylphosphonic acid) complex forms a looser structure in comparison with those for polycarboxylic acids as result of electrostatic repulsion between charged groups.

## 1. Introduction

Interpolymer complexes (IPCs) formed upon the interaction of both synthetic and natural polymers of complex architecture (linear, block and graft copolymers, polymer brushes, stars, liposomes, zwitterions) by means of hydrogen bonds between donor-acceptor units or electrostatic interactions of oppositely charged groups acquire new properties different from the properties of their components. Due to this, they are widely used to create new highly functional composites and materials, substitutes for living tissues, as membrane supersorbents, carriers of medicinal substances [1,2,3].

In recent decades, there has been a considerable interest in the studies of interactions between poly(zwitterions) of various chemical natures and low molecular weight salts, charged polyelectrolytes, and proteins; extensive investigations of the properties of the resulting associates (interpolymer complexes) have been performed [4,5,6,7,8,9,10]. Particularly, this interest is caused by the fact that zwitterionic structures are found in biopolymers. Due to structural similarities between synthetic poly(zwitterions) and cell membranes consisting mainly of zwitterionic phospholipids, the former may be used to develop biocompatible, non-immunogenic materials that will also prevent non-specific protein adsorption [3,5]. These products may also be useful in modifying surfaces of medical products and devices and in membrane technologies.

Poly(zwitterions) are considered as a particular subclass of polyampholytes. While anionic and cationic units in polyampholytes are randomly distributed along the chain, in the case of poly(zwitterions), positively and negatively charged groups are included in the same monomer unit. Therefore, the total charge of the macromolecule containing zwitterions is equal to zero. In general, a zwitterionic polymer is neutral, but a single monomer unit has a high dipole moment (≥15 D). The value of the dipole moment of a poly(zwitterion) depends on the nature of charged groups, the distance between these groups and their mutual arrangement, and the composition and length of the spacer that connects charged groups to the polymer backbone. Strong dipole and electrostatic interactions within zwitterion macromolecules and a high degree of hydration are responsible for many unique properties of poly(zwitterions) [11,12].

The structure of the monomer unit of a poly(zwitterion), nature, and mutual arrangement of its charged groups determines the structure and properties of the formed interpolymer complex. It should be noted that complexes of 1:1 stoichiometry are not necessarily formed. The ability of a zwitterion to form associates with polyelectrolytes is determined mainly by competition between intramolecular interactions in this zwitterion and intermolecular interactions zwitterion–polycation (polyanion), i.e., by the relationship between ionization constants (pK) of polymeric units participating in complex formation. Together with electrostatic interactions, hydrophobic interactions between non-polar groups and hydrogen bonds between donor-acceptor groups of molecules can participate in the formation of poly(zwitterion)-containing associates.

Interactions between poly(zwitterions) and polyelectrolytes can be studied by various methods [7,8,10,13,14,15]. However, there are still virtually no data about zwitterion-containing complexes formed only due to hydrogen bonding and hydrophobic interactions, although contributions of these types of bonds into stabilization of associates with proteins and polyelectrolytes have been mentioned.

In this paper, the method of polarized luminescence (PL) was used to study the interaction of polyzwitterions with polyacids. The PL method involves measurement of the luminescence polarization (*P*) of solutions of luminescent-labeled polymers [16,17,18] and makes it possible to determine nanosecond relaxation times (*τ_IMM_*) that characterize intramolecular mobility of chain fragments or mobility of a macromolecule as a whole [16,17,18].

Measurements of the luminescence polarization in IPC solutions provide an opportunity to study relaxation characteristics of each component included in an IPC; each component, in turn, is labeled with a luminescent marker (luminescent marker is denoted with an asterisk *):

A* + B = (A*B)—relaxation characteristics of the A* component during the complex formation are determined;

A + B* = (AB*)—relaxation characteristics of the B* component during the complex formation are determined.

In addition, PL makes it possible to study relaxation characteristics of interacting macromolecules at all stages of complex formation at various ratios between interacting components in diluted solutions, and it is not necessary to isolate the complex from solution.

In [19,20,21], the PL method was applied to study the structural organization of hydrogen bonded IPC formed by linear macromolecules and macromolecules of complex architecture.

In the present work, PL and IR spectroscopy were used to investigate interactions between poly((2-methacryloyloxyethyl phosphorylcholine) (PMPC) and poly(methacrylic acid) (PMAA), poly(acrylic acid) (PAA), poly(vinylphosphonic acid) (PVPA), copolymers of methacrylic acid (MAA) and 2-deoxy-2-methacrylamido-d-glucose (MAG), and a copolymer of MAG with *N*,*N*,*N*-trimethylaminoethyl methacrylate (TMAEM) methyl sulfate in aqueous solutions. With this purpose, luminescent-labeled and non-luminescent-labeled (“dark”) samples of PMPC, PAA, PMAA, PVPA, and MAG-MAA copolymers were synthesized. Using the PL requires synthesis of luminescent-labeled macromolecules; the content of luminescent markers in a polymer should not exceed 0.1 mol.% in order to avoid side effects, such as excimer formation and migration of electron excitation energy that cause additional depolarization [21]. The *τ_IMM_* values were determined; this parameter characterizes intramolecular mobility of the luminescent-labeled part of macromolecule chain that interacts with a “dark” component in diluted solutions (c ≤ 0.1 wt.%). Possible supramolecular structures of the obtained interpolymer complexes are discussed.

## 2. Materials and Methods

### 2.1. Materials

PMPC was obtained by radical polymerization according to the technique described elsewhere [22].

“Dark” PMAA and PAA were prepared by radical polymerization in *N*,*N*-dimethylformamide (DMFA, Sigma-Aldrich, St Louis, MO, USA) at 60 °C in the presence of azobisisobutyronitrile (Sigma-Aldrich, St Louis, MO, USA) initiator. PVPA was obtained by free-radical polymerization in water solution with 2,2′-azobis-(2-methylpropionamidine) dihydrochloride (Sigma-Aldrich, St Louis, MO, USA) as initiator.

Luminescent-labeled PMPC*, PMAA*, and (MAA-MAG)* copolymers were obtained by radical copolymerization of the corresponding monomers with 9-anthrylmethyl methacrylamide (9-AMA) [22,23,24].

Luminescent-labeled PAA* was prepared by treatment of a “dark” sample with anthryldiazomethane [24].

Polymer synthesis and methods are described in Supplementary Materials.

### 2.2. Methods

Content of acid units in MAG-MAA copolymers was determined by potentiometric titration with a 0.1 N NaOH solution.

The content of luminescent markers (LM) in copolymers was determined by UV-Vis spectroscopy with the use of an SF-256 UVI spectrophotometer (“LOMO Photonika Ltd.”, Saint Petersburg, Russia). The value of the molar extinction coefficient (*ε*) of 9-AMA at 368 nm was equal to 8300 L × cm^−1^ × mol^−1^. In all cases, the contents of LM did not exceed 0.03 mol.%. This excludes the appearance of photophysical processes such as migration of electron excitation energy or formation of excimers that give erroneous values of luminescence polarization.

Molecular masses *M_sD_* of studied samples were determined by the Svedberg relationship:

(1) $$M_{sD}=\left(\frac{s}{D}\right)\times N_{A}\times k\times \frac{T}{\left(1-v\times \rho_{0}\right)}\;$$

where *N_A_* is the Avogadro number, *k_B_* is the Boltzmann constant, *T* is the absolute temperature, *v* is the partial specific volume of macromolecules, and *ρ*_0_ is the solvent density.

Diffusion coefficients *D* were obtained by isothermal translational diffusion using a Tsvetkov polarizing diffusometer(Leningrad University, Leningrad, USSR) [25]. The Lebedev polarizing interferometer (Leningrad University, Leningrad, USSR) was used as an optical system. A glass cuvette of 3 cm long (along the light path) was used. Diffusion coefficients *D* were calculated from the slope of the dependence of the concentration boundary dispersion *σ*^2^ on diffusion time *t*, *D* = *σ*^2^/2*t*. The error of the diffusion coefficient measurements did not exceed 5%.

Sedimentation coefficients (s) of macromolecules in a centrifugal field were measured by the use of a MOM 3180 ultracentrifuge (MOM, Budapest, Hungary) equipped with a polarizing interferometer attachment [25]; the rotation speed of the rotor was 40 × 10^3^ rpm, and the temperature was 24 °C. A two-section cuvette that allows the formation of an artificial boundary was used. Cuvette thickness in the direction of the light beam was 12 mm.

Preparation of solutions. Interaction between PMPC and polyacids was studied in dilute aqueous salt-free solutions. The solution of “dark” (co)polymer (c_pol_ = 0.5 wt.%) was added portionwise to the solution of the luminescent-labeled (co)polymer. The concentration of luminescent labeled solutions of PMAA*, PAA*, P(MAG-MAA_70_)*, and P(MAG-MAA_27_)* was 0.4, 0.3, 0.5, and 1 mg/mL, respectively. It corresponds to the concentration of COOH groups 4.6 × 10^−3^, 4.2 × 10^−3^, 2.6 × 10^−3^ and 1 × 10^−3^ mol/L, respectively. The concentration of luminescent labeled solutions of PMPC* is 0.5 mg/mL, 1.9 × 10^−3^ mol/L.

Polarized luminescence. Polarization of luminescence *P* of the luminescent-labeled polymer *solution* was measured in the steady-state excitation mode using the setup described in [18]. The measurements were carried out in a temperature-controlled cell at 25 °C.

Nanosecond relaxation times *τ_IMM_* that characterize intramolecular mobility (*IMM*) of a polymer chain fragment containing LM [16,17] were determined using the following equation:

(2) $$\tau_{IMM}=\left(\frac{1}{P_{0}^{\prime }}+\frac{1}{3}\right)*3\tau_{fl}/\left(\frac{1}{P}-\frac{1}{P_{0}^{\prime }}\right)$$

where 1/*P*′_0_ is the parameter related to the amplitude of high-frequency movements of the luminescent marker. This parameter was obtained by extrapolation of the linear part of the 1/*P*(*T*/*η*) plot at high *T*/*η* values to *T*/*η*=0; *T* is the absolute temperature (K), *η* is the solvent viscosity (cP); *τ_fl_* is the average lifetime of the excited state of LM bound to the copolymer. The *T*/*η* value was altered by adding saccharose to an aqueous solution of a polymer at a constant temperature. The changes in *τ_fl_* upon adding a viscous component were taken into account when plotting the 1/*P* (*T/η*) dependence. Usually, they did not exceed 10%. Fluorescence lifetime *τ_fl_* of solutions was measured in pulse mode using an LS-100 luminescence spectrophotometer (PTI, Orillia, ON, Canada) at an exciting light wavelength of 358 nm. In all cases, curves of luminescence intensity (*I*) decay were well described by monoexponential dependence *I* = *I*_0_ × *exp(−t/τ)* (the *χ*^2^ parameter is equal to 0.9–1.1).

IR spectroscopy. IR spectra were registered with the use of an IR Fourier spectrometer (Bruker, Ettlingen, Germany) at room temperature in the 400–4000 cm^–1^ range (resolution 4 cm^–1^, number of scans 30); the instrument was equipped with a ZnSe attenuated total reflectance (ATR) micro attachment (Pike). During registration of ATR spectra, correction for depth of light penetration depending on wavelength was made.

## 3. Results and Discussion

**Scheme 1:**
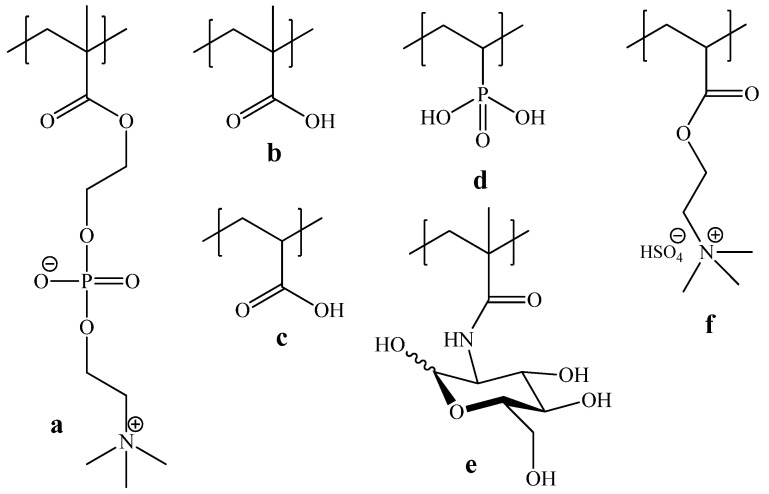
Structures of copolymer units: (**a**) MPC; (**b**) MAA; (**c**) AA; (**d**) VPA; (**e**) MAG; (**f**) TMAEM.

The structures of units of polymers used in this work are presented in Scheme 1.

Table 1 gives characteristics of the polymers used in the present work: their molecular masses, copolymer compositions (the subscript shows the mole percent of ionized groups); for luminescent-labeled (co)polymers, a quantity of (co)polymer units per 1 luminescent marker is given.

### 3.1. Interaction between PMPC and Non-Ionized Poly(carboxylic acids)

Poly(2-methacryloyloxyethyl phosphorylcholine) (PMPC) is a poly(zwitterion); each unit of this polymer contains oppositely charged groups (phosphate anion and quaternary ammonium cation).

Earlier studies of relaxation characteristics of MPC and MAG homopolymers and MPC-MAG copolymers [22] have demonstrated that *τ_IMM_* values found for the copolymers (59 ns) were higher than those for PMPC (35 ns) and PMAG (21 ns). A decrease in the mobility of chain fragments of copolymers (an increase in *τ_IMM_* values) is associated with increasing intramolecular interactions due to the formation of H-bonds between the hydroxyl groups of MAG and the P=O group of MPC. The present work attempts to answer whether the proton acceptor P=O group of MPC can interact with the polymers containing proton donor groups.

**Figure 1:**
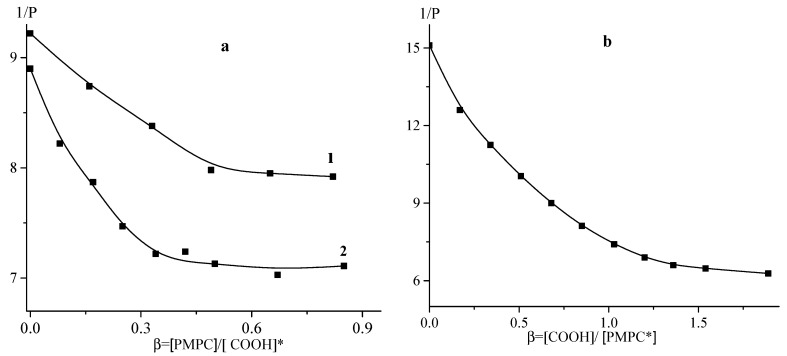
(**a**) Dependences of the 1/P values of an aqueous solution of PMAA* (1) and MAG-MAA_70_* copolymer (2) upon addition of PMPC on the ratio between components β = [PMPC]/[COOH*]; (**b**) dependence of the 1/P value of an aqueous solution of PMPC* upon addition of PMAA on the ratio between components β = [COOH]/[PMPC*].

Figure 1a presents changes in the 1/*P* values of aqueous solutions of PMAA* or (MAG-MAA_70_)* copolymer occurring upon addition of the solution of “dark” PMPC depending on the molar ratio between interacting components β = [PMPC]/[COOH]. Figure 1b illustrates the corresponding dependence for an aqueous solution of PMPC* upon adding the solution of “dark” PMAA. It is seen in Figure 1a,b that the 1/*P* values decrease in both cases upon the addition of “dark” components. These changes indicate interactions between labeled molecules and the added “dark” components.

**Figure 2:**
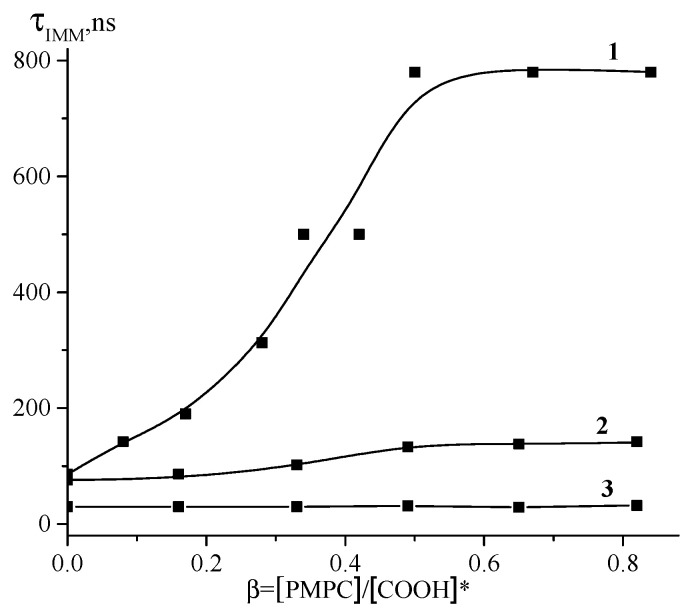
Dependences of *τ_IMM_* of PMAA* (1), copolymer (MAG-MAA_70_)* (2), copolymer (MAG-MAA30)* (3) on the ratio β = [PMPC]/[COOH].

**Figure 3:**
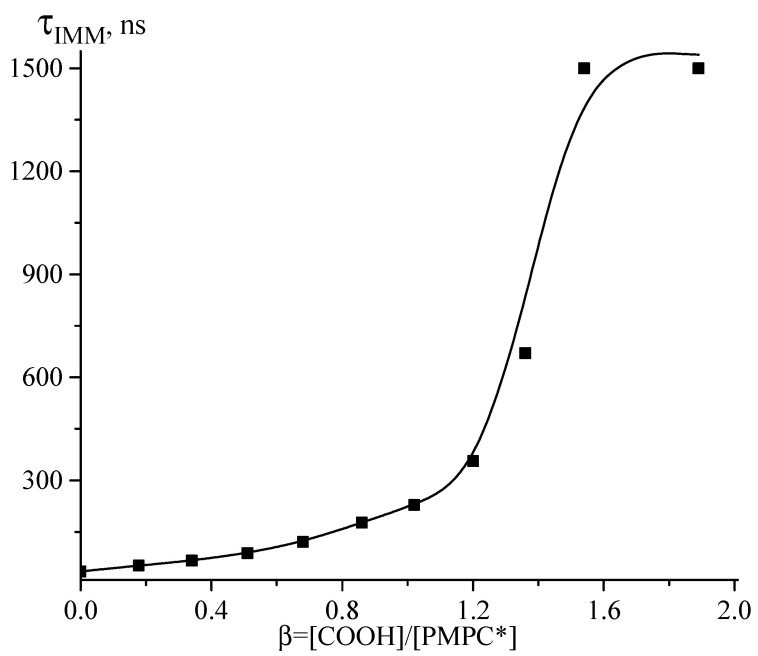
Dependence of *τ_IMM_* of PMPC* on the ratio β = [COOH]/[PMPC]* upon addition of a solution of “dark” PMAA.

Figure 2 presents dependences of relaxation times *τ_IMM_* of PMAA* and copolymers (MAG-MAA_70_)* (1) on β. Figure 3 presents the dependence of *τ_IMM_* of PMPC* on β. In both cases, values of relaxation times increased, which indicated a decrease in intramolecular mobility of interacting components, which is caused by the formation of intermolecular associates and an increase in the local concentration of units in these associates.

The *τ_IMM_* values for PMAA* increase from 86 to 780 ns. The *τ_IMM_* values for PMPC* increase more significantly (from 35 to ~1500 ns). Analysis of variations in intramolecular mobilities of each component of a multicomponent polymer-based system in dilute solution provides information about the structural organization of the resulting complex. It has been demonstrated [26] that the formation of an interpolymer complex between complementary linear macromolecules led to noticeable growth in the *τ_IMM_* values by 1–2 orders of magnitude). Mobility of chains in IPC becomes virtually similar, while mobilities of the same chains in their free states may differ considerably; this fact is an indirect proof of the formation of extended two-strand structures.

Significant differences between the *τ_IMM_* values of PMAA* and PMPC* macromolecules in the associate indicate that, in this case, the formation of two-strand structures between components is unlikely.

Some differences in the behavior of the *τ_IMM_*(β) dependences for [PMPC]/[PMAA*] and [PMAA/[PMPC*] are to be noted. For the [PMPC]/[PMAA*] system, the *τ_IMM_* curve reaches a plateau at β = 0.5. For the [PMAA]/[PMPC*] system, *τ_IMM_* of PMPC* rises slowly from 35 to 300 ns until the β value reaches 1.0; then, in the range of β values from 1.0 to 1.9, nanosecond relaxation time increases sharply from 300 to ≥1500 ns, and the curve reaches a plateau at β = 1.6.

With a knowledge of the β values corresponding to the beginning of a plateau in the *τ_IMM_*/(β) plot, it is possible to determine the ratio between interacting groups in an associate. The lowest mobility of PMAA* chain fragments is observed when the complex contains one MPC unit per two MAA monomer units. This result indicates that a portion of MAA units exists in a free non-bound state; apparently, the formation of the equimolar complex is hindered unlike interaction between zwitterions and polyelectrolytes or proteins [4]. The lowest mobility of PMPC* chain fragments is observed when 1.5 ÷ 1.6 PMAA units interact with one PMPC unit (3:2). Taking into account high *τ_IMM_* values for PMPC* and the fact that they are reached at the lower ratio between interacting groups, it can be suggested that PMPC chains form a “core” of the associate, and PMAA chains surround this core.

Studies of the (MAG-MAA)* copolymers containing 70 and 26 mol.% of MAA units revealed that, upon the interaction between this copolymer and PMPC, the *τ_IMM_* values for the labeled copolymer (MAG-MAA_70_)* increased from 80 to 150 ns, while, for the (MAG-MAA_30_)* copolymer, no changes were observed (Figure 2, curves 2 and 3, respectively). Since the total concentration of MAA units in all cases was the same, this difference is caused by (i) forming a comparatively low number of contacts per 1 macromolecule and (ii) steric hindrances that prevent the inclusion of bulky side MAG moieties into an associate. An additional factor that prevents efficient interaction between PMPC and (MAG-MAA) copolymers is the high hydrophilicity of the interacting components. Thus, the hydration shell of one PMPC unit contains up to 15 water molecules [27].

**Figure 4:**
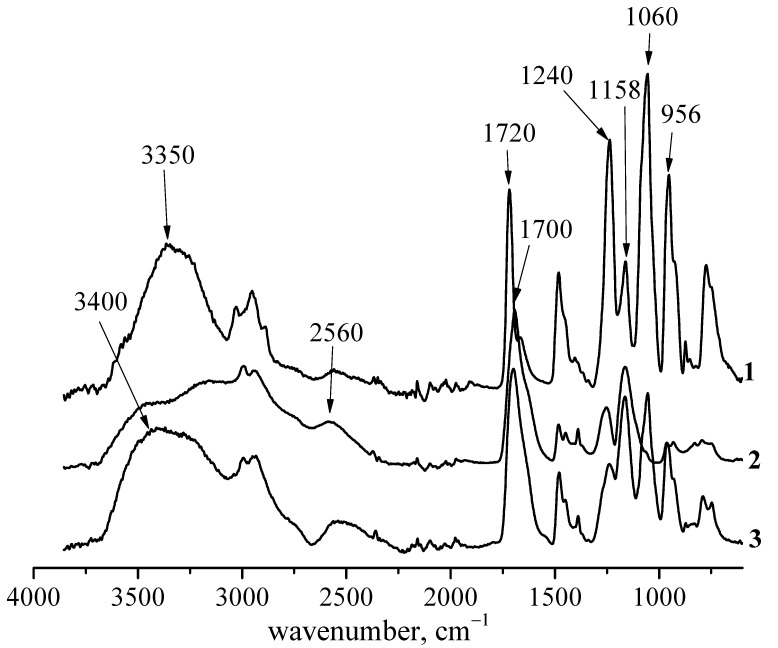
IR-ATR spectra of PMPC (1), PMAA (2), and their associate (3).

The obtained associates were characterized by IR spectroscopy. Figure 4 presents IR-ATR spectra of PMPC (1), PMAA (2), and their complex (3).

An intense band at 1240 cm^−1^ in the PMPC spectrum is attributed to vibrations of P=O groups. The shift of the P=O vibration band in the PMPC spectrum from the position typical of the P=O groups of alkylphosphoric acid esters (1290–1260 cm^–1^ in the spectra of organophosphorus compounds) is caused by the inductive influence of substituents on the type of P=O bond and formation of a zwitterion with a high dipole moment (~15 D). The band in the 1170–1150 cm^−1^ region is assigned to vibrations of the P–O–C_2_H_4_-group. The vibration band of the P–O–C-group is also observed in the 1060–990 cm^−1^ area [28].

The frequencies of stretching vibrations of polymer groups and their changes upon the formation of complexes are shown in Table 2.

**Figure 5:**
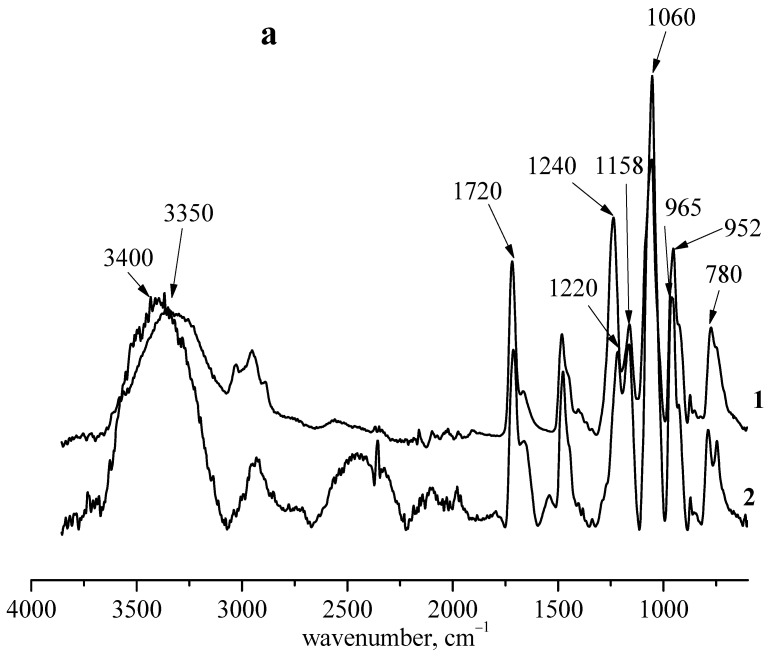

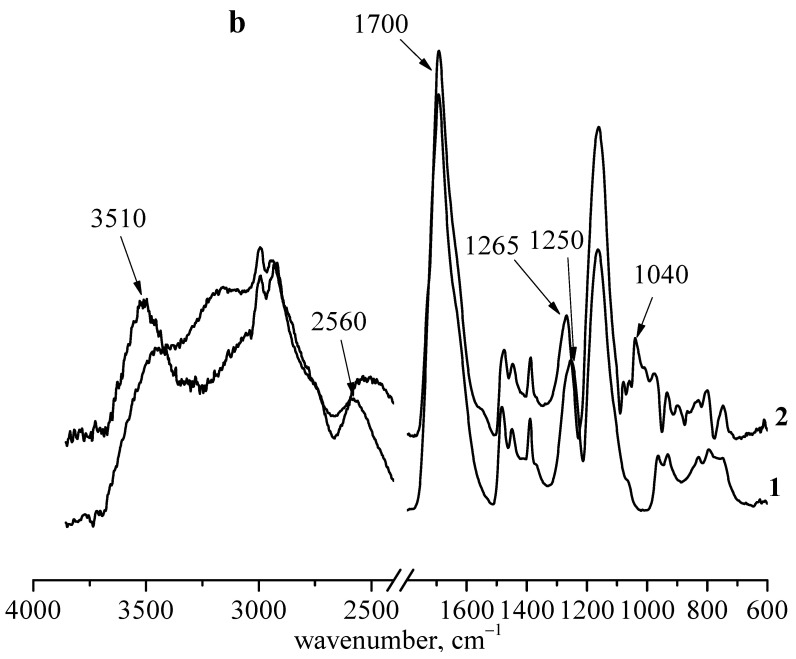
(**a**) IR spectrum of PMPC (1) and the difference spectrum obtained by subtracting the PMAA spectrum from the associate spectrum (2); (**b**) IR spectrum of PMAA (1) and the difference spectrum obtained by subtracting the PMPC spectrum from the associate spectrum (2).

With the purpose of a more detailed investigation of the IR data, the difference spectra were obtained by subtracting spectra of components from the spectrum of the associate (Figure 5a,b).

Comparison of the difference spectrum obtained by subtracting PMAA spectrum from the spectrum of the complex and the pure PMPC spectrum shows that the vibration band of free P=O groups is shifted from 1240 cm^−1^ to 1220 cm^−1^, i.e., toward the position of the P=O groups participating in the formation of hydrogen bonds. Comparison of the difference spectrum obtained by subtracting the PMPC spectrum from the spectrum of the complex and the spectrum of pure PMAA involved in complex formation reveals a decrease in the band’s intensity at 2560 cm^−1^ that corresponds to valence vibrations of the bound OH group of the carboxylic fragment. In addition, a new peak at 3510 cm^−1^ appears; this band is typical of vibrations of less firmly bound OH groups. The observed changes in difference spectra may be explained by the substitution of the bond between OH and –C=O in the PMAA carboxylic fragment for the bond between OH and –P=O group of PMPC that has lower bond energy [28].

In addition, no significant shifts of the peaks attributed to the groups forming zwitterion (ionic pair) were registered in the IR spectrum of the complex; therefore, it is concluded that they do not participate in complex formation. Thus, it may be assumed that associates between PMPC and non-charged PMAA macromolecules are formed due to hydrogen bonds between the P=O group of PMPC and the OH group of PMAA carboxylic group.

Since COOH groups form a hydrogen bond with the P=O group and terminal –N^+^(CH_3_)_3_ groups become shielded from solvent molecules, the associates become more hydrophobic. As a result, an increase in the concentration of interacting macromolecules leads first to opalescence and then (at c ~1 wt.%) to the appearance of a precipitate.

**Figure 6:**
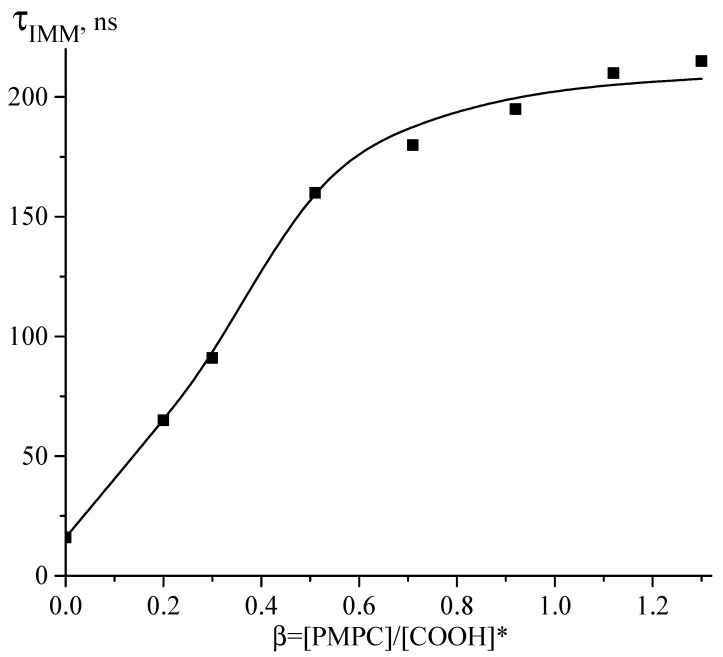
Dependence of *τ_IMM_* of PAA* on the ratio β = [PMPC]/[COOH]*.

Interaction between PAA* and PMPC is also accompanied by a decrease in the 1/*P* of PAA* solution and an increase in the *τ_IMM_* value upon addition of PMPC to the solution of the polyacid (Figure 6).

Relaxation times *τ_IMM_* increase from 16 to 220 ns, i.e., by more than one order of magnitude. The maximum values of *τ_IMM_* are reached at β ~ 0.9; i.e., 10 PAA units per ~9 PMPC units are involved in complex formation. Comparing this complex with the PMPC-PMAA system shows significant differences in the maximum *τ_IMM_* values and composition of associates. These differences in the *τ_IMM_* values of polyacids included in the complex (230 ns for PAA and 780 ns for PMAA) and the number of COOH groups per 1 MPC unit are caused by the influence of α-methyl groups on the structure of the associate. It is known that, in aqueous solutions, local compact structures appear in the linear PMAA macromolecules due to interaction between an α-methyl groups; accordingly, lower intramolecular mobility of PMAA chains is registered (as compared to that of PAA chains) [29,30] Thus, it may be supposed that hydrophobic interactions between the α-methyl group of PMAA and non-polar groups of the PMPC unit play an essential role in forming the PMPC-PMAA complex.

**Scheme 2:**
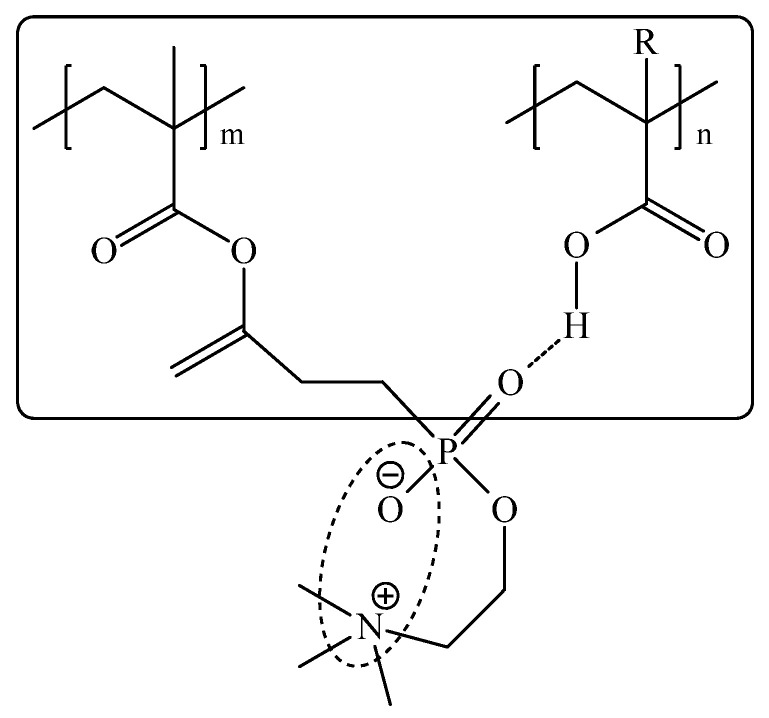
Possible structure of the fragment of the complex between PMPC and non-ionized PAA (PMAA). Solid line: fragments of macromolecules forming the associate; dotted line: zwitterion.

The possible structure of the fragment of the complex between PMPC and non-ionized PAA or PMAA is presented in Scheme 2:

### 3.2. Interaction between PMPC and Ionized Poly(carboxylic acids)

In PMPC macromolecules, anionic and cationic groups are charged virtually at all pH values. Hence, these groups in zwitterions stay fully deprotonated over a broad pH range. It was revealed that the 1/*P* values and the *τ_IMM_* values remain virtually the same both upon the addition of solutions of ionized “dark” PAA or PMAA to an aqueous solution of PMPC* and, conversely, upon addition of a solution of “dark” PMPC to aqueous solutions of luminescent-labeled ionized PAA* or PMAA*. This result indicates the absence of interactions between macromolecules. At the same time, upon addition of luminescent-labeled copolymer (MAG-TMAEM47)* (where TMAEM units serve as a model for PMPC terminal group) to the solution of PMAA, the *τ_IMM_* values increase by more than 1.5 orders of magnitude from 35 to ≥1000 ns, which indicates the formation of interpolyelectrolyte complex.

The obtained results indicate that, in aqueous solutions, weak poly(carboxylate anions) cannot compete with a phosphate group (strong acid), which forms an ionic pair with a cationic group. Theoretical calculations [31] also show that carboxylic groups are not competitive with phosphates in the formation of strong complexes with cationic groups such as quaternary or tertiary amino groups). This phenomenon was observed experimentally in [10] using fluorescence titration (quenching) of PMAA with poly(carboxybetaine).

### 3.3. Interaction between PMPC and Poly(vinylphosphonic acid)

Poly(vinylphosphonic acid) contains two OH groups capable of ionization. It has been demonstrated [31,32] that, due to electrostatic interactions between geminal charges and the formation of intramolecular hydrogen bond between OH and P=O groups, dissociation of the second proton in the phosphonic unit became impossible. Therefore, PVPA should be considered a monobasic acid of a medium strength [31,32], unlike PMAA and PAA, which are classed as weak polyacids. Thus, in an aqueous solution, each monomer unit of PVPA contains three functional groups: negatively chargeable –O– group, proton donor –OH group, and proton acceptor P=O group. These fragments can form the associates with macromolecules containing positively charged groups, proton acceptor groups, or proton donor ones.

**Figure 7:**
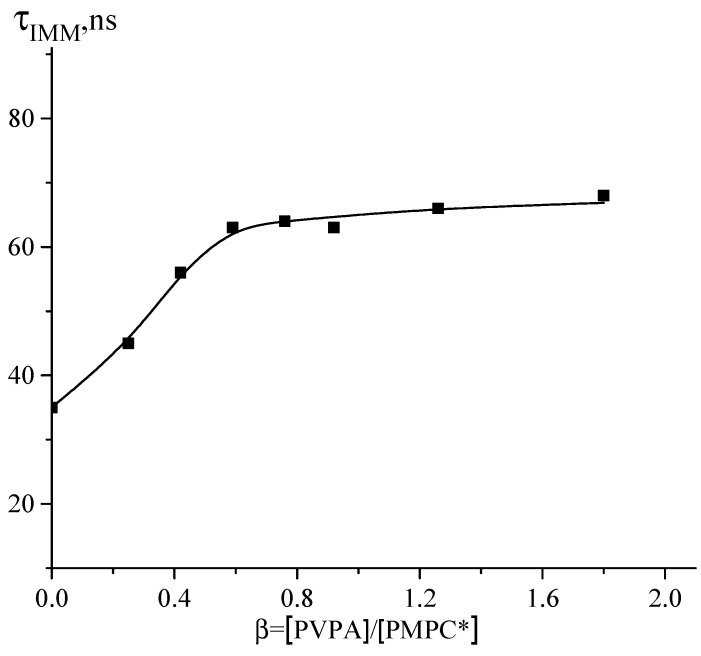
Change in *τ_IMM_* characterizing mobility of PMPC* chain fragments upon addition of PVPA to its solution.

Figure 7 shows the change in *τ_IMM_* values of PMPC* upon adding PVPA to its solution. It is seen that the *τ_IMM_* values characterizing mobility of PMPC* chain fragments rise with increasing of the [PVPA]/[PMPC*] ratio (β) as in the case of the PMAA/PMPC* system. However, the limiting-reached value of relaxation time *τ_IMM_* = 68 ns is more than an order of magnitude lower than the corresponding values for the PMAA/PMPC*complex.

The possibility of forming a polyelectrolyte complex between poly(vinylphosphonic acid) and PMPC is very low. This conclusion can be drawn from the studies of the interaction between PVPA and a model copolymer (MAG-TMAEM)*. Upon mixing aqueous solutions of PMPC and (MAG-TMAEM)* copolymer, already at the ratio [PMPC]:[(MAG-TMAEM)*]=1:10, a precipitate is formed. This precipitate dissolves in 0.1 N NaCl. The result indicates efficient interaction between the PMPC and (MAG-TMAEM)* macromolecules and the formation of interpolyelectrolyte complex under these conditions. In the PVPA and PMPC* system, PVPA cannot compete with the phosphate group of the ionic pair for interaction with the quaternary nitrogen atom, although it is a stronger acid than PMAA. Thus, it may be concluded that an increase in *τ_IMM_* of PMPC* upon interaction with PVPA is related to the formation of hydrogen bonds between P=O group of PMPC and non-ionized HO–P(=O)(O^−^) group of PVPA. Significantly lower values of relaxation times *τ_IMM_* indicate that local motions in PMPC* macromolecules upon formation of associate with PVPA are not “frozen” in contrast to associates with PMAA (PAA), which is indicative of the formation of “loose” associates. Electrostatic repulsion between charged groups ^−^O–P(=O)(OH) in PVPA prevents compactization of PVPA-PMPC complex (unlike complexes of PMPC with PMAA or PAA). In addition, the appearance of this structure is caused by the formation of the P=O…HO–P(=O)–O^−^ bond that has lower energy than the P=O…HOOC bond.

## 4. Conclusions

The studies of relaxation parameters of luminescent-labeled polymers by polarized luminescence demonstrated that interaction between PMPC and non-ionized carboxylic polyacids in diluted aqueous solutions leads to the formation of interpolymer complexes; intramolecular mobility of the macromolecules included in the associate decreased significantly (3–30 times). Hydrogen bonds play a significant role in complex formation; hydrophobic interactions also participate in this process. The formation of complexes was confirmed by IR spectroscopy. Interaction between PMPC and PVPA leads to more “loose” associates compared to PMPC/PMAA or PMPC/PAA complexes. This result is due to electrostatic repulsion between charged groups of PVPA. The data obtained expand the opportunities of polyzwitterions modification for their application in various technological processes in industry, biotechnology, and medicine.

## Figures and Tables

**Table 1:** Characteristics of the studied polyelectrolytes (copolymer composition, MM, and amount of (co)polymer units per 1 luminescent marker).

(co)polymer	MM × 10^−3^	[(co)polymer unit]:[LM]
PMPC	463	
PMPC *	280	450
PMAA	306	
PMAA *	267	670
Copolymer (MAG-MAA_70_) *	67	420
Copolymer (MAG-MAA_26_) *	97	600
PAA	130	
PAA *	130	300
PVPA	31	
Copolymer (MAG-TMAEM_47_) *	65	380

* luminescent-labeled macromolecules.

**Table 2:** Stretching vibrations of polymer groups and their changes upon the formation of complexes.

Functional Group	ν, cm^−1^ Polymer	ν, cm^−1^ Complex
OH	3400, 3500	No change
Bonded OH	2560	Intensity decrease
COOH	1700	No change
COO– (ester)	1720	Broaden
P=O	1240	1220
P–O–C2H5	1170–1150	No change
P–O–C	1060–990	1040
P–O–C	965	952

## Supplementary Materials

The following are available online at https://www.mdpi.com/article/10.3390/polym14030407/s1.
