# Supplementary material for: Interpolymer Complexes of Poly(methacryloyloxyethyl phosphorylcholine) and Polyacids
Source: Polymers (Basel). 2022 Jan 20;14(3):407. doi: 10.3390/polym14030407 (PMC8839767; doi:10.3390/polym14030407)
Supplement: Supplementary file 1 [file polymers-14-00407-s001.zip › polymers-1520033-supplementary.pdf]

Supplementary Materials

Synthesis of (co)polymers

Reactions of free radical (co)polymerization were carried out in sealed ampoules in argon atmosphere at 60 °C in N,N-dimethylformamide (DMFA), CH_3OH or water solutions with 2,2-azobisisobutironitrile (AIBN) or 2,2'-azobis-(2-methylpropionamidine) dihydrochloride (AMP) as an initiator for 24 h. Antryldiazomethane (ADM) or 9-anthrylmethyl methacrylamide (9-AMA) were used as luminescent labels. Then the copolymer solution was purified from low molecular weight impurities by dialysis against water. Spectra/Por 7 dialysis membranes (Spectrum Laboratories, Inc., USA) that allow removing substances with molecular masses MM ≤1000 were used. The final product was isolated by lyophilization. Molecular masses were estimated by measuring sedimentation and diffusion.

1. **"Dark" and luminescent-labeled PMPC homopolymer**

Polymerization conditions
4.6 ml DMFA
0.5 g PMPC (0.00169 mol.), C = 0.037 mol/l
0.012 g 9-AMA (0.0000044 mol.), C = 0.000095 mol/l
0.01 g AIBN (0.000061 mol.), C = 0.00132 mol/l
Yield
0.426 g (85 %)
Polymer
MM = 280,000

Polymerization conditions
3 ml DMFA
0.5 g PMPC (0.00169 mol.), C = 0.0563 mol/l
0.05 g AIBN (0.0000305 mol.), C = 0.001 mol/l
Yield
0.435 g (87 %)
Polymer
MM = 463,000

2 **"Dark" and luminescent-labeled PMAA homopolymer**

5 ml DMFA
1.0 g MAA (0.0116 mol.), C = 2.3 mol/l
0.01 g AIBN (0.00006 mol.), C = 0.012 mol/l
Yield
0.89 g (89 %)
Polymer
MM = 306,000

5 ml DMFA
1.0 g MAA (0.0116 mol.), C = 2.3 mol/l
0.01 g AIBN (0.00006 mol.), C = 0.012 mol/l
0.012 g 9-AMA (0.0000044 mol.), C = 0.000095 mol/l
0.01 g 9-AMA (0.000036 mol.), C = 0.0073 mol/l
Yield

0.84 g (84 %)
Polymer
MM = 267,000

3 "Dark" PAA homopolymer

5 ml DMFA
0.83 g AA (0.0115 mol.), C = 2.3 mol/l
0.03 g AIBN (0.00018 mol.), C = 0.036 mol/l
Yield
0.75 g (90 %)
Polymer
MM = 130,000

Luminescent-labeled PAA homopolymer

Reaction conditions
10.0 ml CH_3OH
0.05 g PAA (0.0007 mol.), C = 0.07 mol/l
0.06 g ADM (0.000028 mol.), C = 0.0000028 mol/l

4 "Dark" PVPA homopolymer

Polymerization conditions
0.1 ml H_2O
0.5 g VPA (0.0046 mol.), C = 4.6 mol/l
0.005 g AMP (0.000018 mol.), C = 0.18 mol/l
Yield
0.269 g (54 %)
Polymer
MM = 31,000

5 Luminescent-labeled MAG-MAA copolymers

Copolymerization conditions
2.5 ml DMFA
0.3 g MAG (0.00121 mol.), C = 0.0178 mol/l
0.5919 g MAA (0.0069 mol.), C = 0.2752 mol/l
MAG : MAA = 18 : 82 mol.%
0.0223 g 9-AMA (0.000081 mol.), C = 0.0032 mol/l
0.0178 g AIBN (0.00011 mol.), mol/l 0.004 mol/l
Yield
0.707g, 79%
Copolymer
MAG : MAA =30 : 70 mol.%

Copolymerization conditions
5.0 ml DMFA
0.7 g MAG (0.0028 mol.), C = 0.0178 mol/l
0.1045 g MAA (0.0012 mol.), C = 0.024 mol/l
MAG : MAA = 70 : 30 mol.%

0.0111 g 9-AMA (0.00004 mol.), C = 0.00008 mol/l
0.0161 g AIBN (0.000098 mol.), 0.0019 mol/l
Yield
0.700g, 87%
Copolymer
MAG : MAA = 74 : 26 mol.%

Compositions of the copolymers were determined by potentiometric titration of MAA units with 0.1 N NaOH solution in 0.1 N NaCl solution

6 Luminescent-labeled MAG-TMAEM copolymer

Copolymerization conditions
8.0 ml H_2O
0.6 g MAG (0.00243 mol.), C = 0.030 mol/l
0.458 g TMAEM (0.00162 mol.), C = 0.02 mol/l
MAG : TMAEM = 60.0 : 40.0 mol.%
0.0111 g 9-AMA (0.0000364 mol.), C = 0.00045 mol/l
0.02 g AMP (0.00012 mol.), 0.00153 mol/l
Yield
0.62 g, 59 %
Copolymer
MAG : TMAEM = 53.0 : 47.0 mol.%

Compositions of the copolymers were determined by evaluating of sulfur content by elemental analysis.

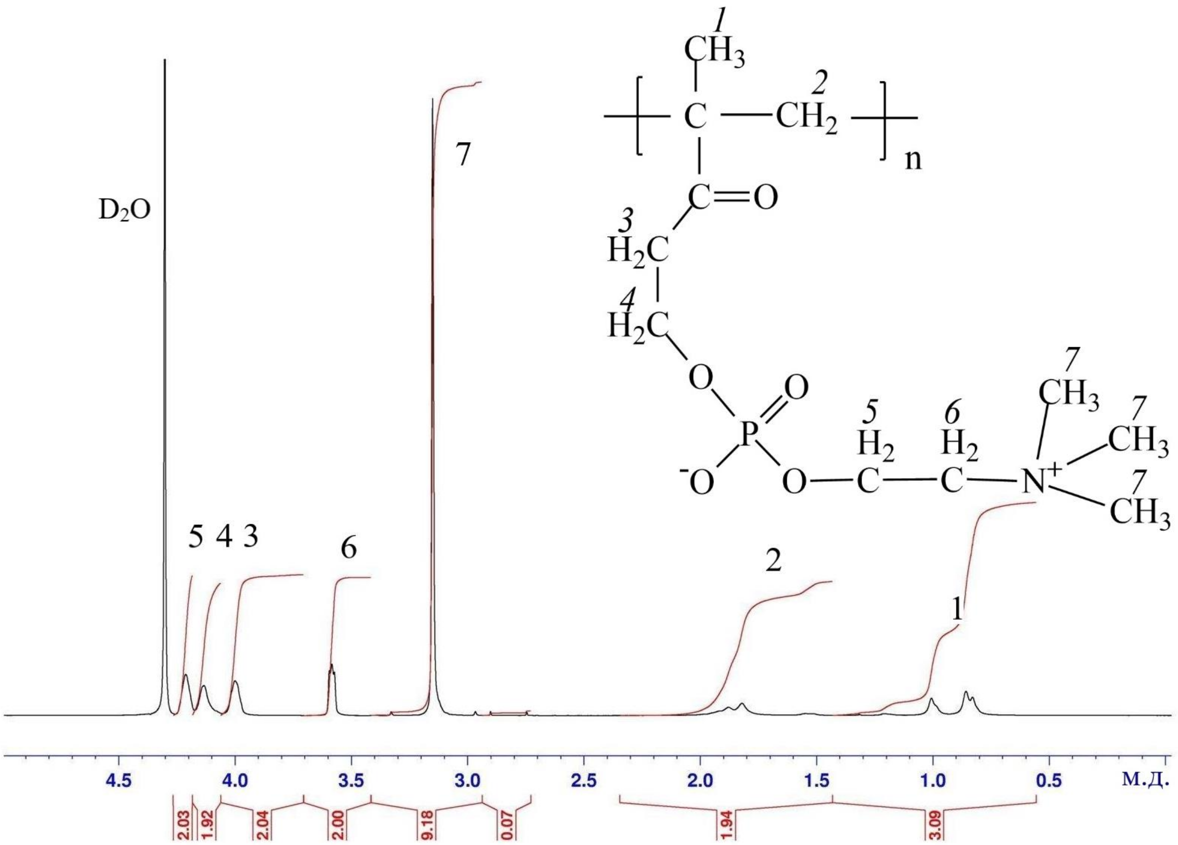

^{1}H NMR-spectra of **PMPC**.

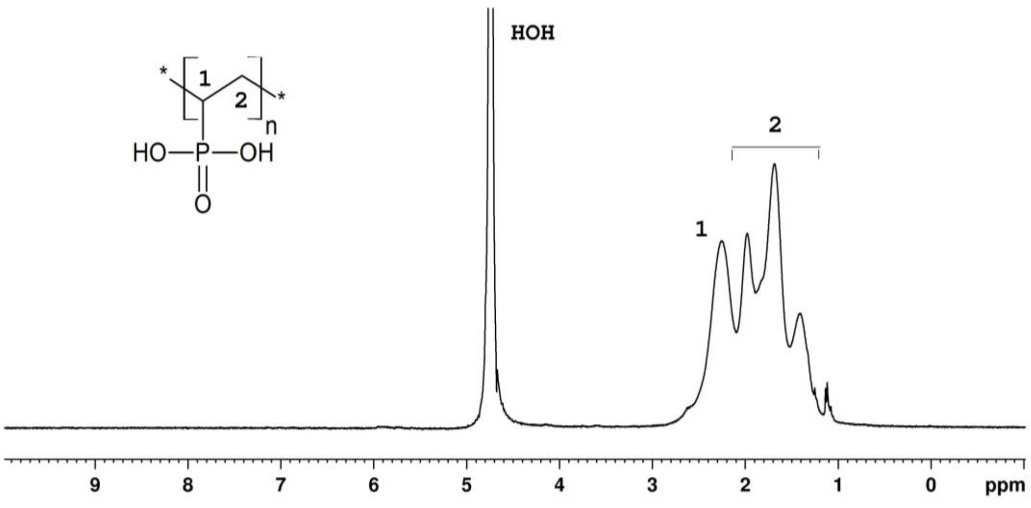

^{1}H NMR-spectra of **PVPA**
